# Genome-wide identification and functional analysis of dysregulated alternative splicing profiles in sepsis

**DOI:** 10.1186/s12950-023-00355-w

**Published:** 2023-09-25

**Authors:** Dilixiati Tuerdimaimaiti, Buzukela Abuduaini, Shaotao Kang, Jinliang Jiao, Mengchen Li, Wolazihan Madeniyati, Baihetinisha Tuerdi, Gulisitan Aili, Reyila Tuerhong, Ajiguli Kulaxi

**Affiliations:** 1 Department of RICU, The First Affiliated Hospital of Xinjiang Medical University, 393 South Li Yu Shan Road, Wulumuqi, Xinjiang 830054 China; 2 The Intensive Care Unit, The First Affiliated Hospital of Xinjiang Medical University, Wulumuqi, Xinjiang 830054 China

**Keywords:** Alternative splicing, Sepsis, Apoptosis, RBP, Immune effector cells

## Abstract

**Background:**

An increasing body of evidence now shows that the long-term mortality of patients with sepsis are associated with various sepsis-related immune cell defects. Alternative splicing (AS), as a sepsis-related immune cell defect, is considered as a potential immunomodulatory therapy target to improve patient outcomes. However, our understanding of the role AS plays in sepsis is currently insufficient.

**Aim:**

This study investigated possible associations between AS and the gene regulatory networks affecting immune cells. We also investigated apoptosis and AS functionality in sepsis pathophysiology.

**Methods:**

In this study, we assessed publicly available mRNA-seq data that was obtained from the NCBI GEO dataset (GSE154918), which included a healthy group (HLTY), a mild infection group (INF1), asepsis group (Seps), and a septic shock group (Shock). A total of 79 samples (excluding significant outliers) were identified by a poly-A capture method to generate RNA-seq data. The variable splicing events and highly correlated RNA binding protein (RBP) genes in each group were then systematically analyzed.

**Results:**

For the first time, we used systematic RNA-seq analysis of sepsis-related AS and identified 1505 variable AS events that differed significantly (*p* <= 0.01) across the four groups. In the sepsis group, the genes related to significant AS events, such as, *SHISA5* and *IFI27*, were mostly enriched in the cell apoptosis pathway. Furthermore, we identified differential splicing patterns within each of the four groups. Significant differences in the expression of RNA Binding Protein(RBP) genes were observed between the control group and the sepsis group. RBP gene expression was highly correlated with variant splicing events in sepsis, as determined by co-expression analysis; The expression of DDX24, CBFA2T2, NOP, ILF3, DNMT1, FTO, PPRC1, NOLC1 RBPs were significant reduced in sepsis compared to the healthy group. Finally, we constructed an RBP-AS functional network.

**Conclusion:**

Analysis indicated that the RBP-AS functional network serves as a critical post-transcriptional mechanism that regulates the development of sepsis. AS dysregulation is associated with alterations in the regulatory gene expression network that is involved in sepsis. Therefore, the RBP-AS expression network could be useful in refining biomarker predictions in the development of new therapeutic targets for the pathogenesis of sepsis.

## Introduction

Pre-messenger RNA splicing has been reported to be a critical step in RNA maturation, which includes joining exons together as well as removing introns. The process of splicing is accomplished in the cell nucleus by means of one of two different macromolecular ribonucleoprotein complexes, which are called the major and minor spliceosomes [1]. It has been determined that greater than 90% of the genes that are expressed in humans undergo alternative splicing [2], allowing individual genes to give rise to several different mRNAs that encode unique proteins, which can significantly expand the proteome complexity.

Published reports have described numerous types of AS events, although cassette exons appear to be the dominant form. Regulation of AS events is known to occur in a spatiotemporal-dependent manner [1] through combined actions of cis-elements as well as trans-factors [3]. Furthermore, aberrant splicing events have been associated with numerous diseases [4, 5].

Dysregulation of the host’s response to infections appears to be the primary cause of sepsis and represents a leading cause of morbidity, mortality, and healthcare utilization for intensive care unit (ICU) patients [6]. The pathogenesis of sepsis is relatively complex. However, while there are many related studies on the pathogenesis of sepsis, the precise mechanisms involved have yet to be elucidated. In particular, the lack of special treatment methods for sepsis is closely associated with high levels of mortality. Studies that have been carried out recently have demonstrated that the transcriptome signatures of the host are able to distinguish between causes of sepsis that are viral and those that are bacterial. RNA-seq analysis of responses mounted in whole blood by the immune system of the host could reveal the “true” prevalence and epidemiology underlying the occurrence of sepsis [7]. Several studies have indicated that AS dysregulation is associated with several clinical entities, including cancer, by influencing cell proliferation, apoptosis, invasion, migration, and metabolism [8]. The diversity and flexible characteristics of proteins are critical in regulating AS and are a prerequisite for the maintenance of functional immune responses. Numerous genes that are involved in signaling in the innate or adaptive immune pathways are known to undergo varying degrees of AS [9]. Based on the functions of the genes that are spliced, AS can influence the physiological functions that the immune system carries out in a range of ways. In addition, alterations in the mechanisms by which splicing occurs and even participation by non-coding RNAs may result in alterations in the patterns of splicing that occur in sepsis-related genes [10]. However, there is a lack of research on the role of AS with regard to the immune regulation of sepsis and the progression of this condition. Therefore, the identification of potential targets for AS could provide information that might prove beneficial in developing novel methods that could be used in diagnosing and treating sepsis.

In this investigation, we profiled mRNAs that were differentially expressed (DE) in sepsis, which was accomplished through the assessment of a publicly available RNA-seq dataset and the construction of a new co-expression network for DE mRNAs. The variable splicing events and highly correlated RNA binding protein (RBP) genes in differentially expressed (DE) mRNAs were then comprehensively analyzed. Differentially expressed analysis was then utilized in the identification of differentially expressed alternative splicing (DEAS) events among sepsis, septic shock, and healthy individuals. In addition, correlations that occurred between the DEAS events and immune features or functional analyses were assessed. Cluster analysis, which was based on DEAS, was used to accurately represent any differences that were observed among the included study groups with respect to the immune microenvironment. We also predicted the target genes and related RNA binding proteins (RBP) and determined their functionality. Various genes with different alternative splicing types were chosen to confirm the alternative splicing events that were identified using bioinformatic analysis. This step was followed by experimentally verifying the splice variants that were predicted by using reverse transcription-polymerase chain reaction (RT-PCR). The results of this study will contribute to a deeper and more inclusive understanding of how AS is involved in the process of sepsis. Our findings also might be useful in identifying novel therapeutic targets that could help decrease patient morbidity and mortality resulting from septic syndromes.

## Material and methods

### Accessing and processing publicly available data

The publicly available sequence data files from GSE154918 [11] were accessed from the Sequence Read Archive (SRA). Then the SRA Run files were changed to the fastq format by the NCBI SRA Tool fastq-dump (v.2.8.0). The FASTX-Toolkit (v.0.0.13; http://hannonlab.cshl.edu/fastx_toolkit/) was utilized to trim the data by removing low-quality bases(Remove the base with terminal mass less than 20; Remove 30% of the reads whose base mass is less than 20) in raw reads.

### The alignment of the reads and differentially expressed gene (DEG) analysis

The processed reads underwent alignment to the human GRch38 genome via HISAT2(v.2.2.1) [12]. Uniquely mapped reads were screened for further analysis. We then calculated the reads number located on each gene. The gene expression levels were evaluated with FPKM (fragments per kilobase of exon per million fragments mapped). DEseq2 (v. 1.30.1) software was used to perform differential gene expression analysis using the reads count file [13]. DEseq2 was then utilized to assess the differential expression that occurred among two or more samples to decide if a specific gene was differentially expressed through the calculation of fold changes (FC) as well as determination of the false discovery rate (FDR).

**Two critical parameters were determined**

1. FC: fold change, which indicated the absolute ratio of the change in expression.
2. FDR: the false discovery rate.

**The criteria used to assess the significant difference in expression included the following:**

- FC ≥ 2 or ≤ 0.5, FDR ≤ 0.05;

### Analysis of alternative splicing

The alternative splicing events (AS) and regulated alternative splicing events (RAS) that were observed among the different groups in this study were defined and measured with the SUVA (v2.0)(Splicing site Usage Variation Analysis) pipeline, as described previously [14]. The proportion of reads with a SUVA AS event (pSAR) was determined for each AS event.

### Co-expression analysis

A co-disturbed network was constructed utilizing 79 samples obtained from the GSE154918 dataset. The network was constructed between the RBP expression and the splicing ratio of the RAS events (pSAR >= 50. Next, the Pearson’s correlation coefficients (PCCs) were determined between these parameters, which allowed the classification of their relationship into one of three classes: positive correlation, negative correlation, or no correlation, as determined by the PCC value. Factors with a Pearson’s correlation of 0.7 or greater and with a *P*-value equal to or less than 0.01 were retained.

### Analysis of functional enrichment

To comprehensively identify the different DEG functional categories, we performed Gene Ontology (GO) and KEGG pathway analysis of the splicing data utilizing a KOBAS 2.0 server [15]. Using KOBAS (http://kobas.cbi.pku.edu.cn/), the hypergeometric test and the Benjamini-Hochberg FDR controlling procedure were performed to assess each term’s enrichment. Next, we performed biological process pathway analysis by Reactome (http://reactome.org) to identify the functional enrichment of selected genes. The KEGG pathways, as well as the Gene Ontology (GO) terms, were identified and used to define the DEG functional categories.

### RNA extraction and real-time quantitative PCR

Peripheral blood of 20 clinical specimens was collected, including 5 cases in the mild INF1 group, 5 cases in the Seps group, 5 cases in the Shock group, 5 cases in the HLTY, and the inclusion criteria of the sepsis group were strictly in accordance with the international sepsis diagnostic standards. This study was reviewed and approved by the Ethics Committee of the First Affiliated Hospital of Xinjiang Medical University (*Approval Number K202306-09*). cDNA synthesis was done by reverse transcription kit(R323-01, Vazyme, China) at 42˚C for 5 min, 37 ˚C for 15 min, 85 ˚C for 5 s performed on the thermocycler(T100, Bio-Rad, USA). And qPCR was performed on the ABI QuantStudio 5, followed by denaturing at 95˚C for 10 min, 40 cycles of denaturing at 95˚C for 15 s and annealing and extension at 60˚C for 1 min. Each sample had three technical replicates. The concentration of each transcript was then normalized to GAPDH (glyceraldehyde-3-phosphate dehydrogenase) and mRNA level using 2- ΔΔCT method to analysis (Livak and Schmittgen 2001). Comparisons were performed with the two-way ANOVA or the paired Student’s t-test by using GraphPad Prism software (Version number8.0, San Diego, CA).

### Statistical analysis

Principal component analysis (PCA) analysis was completed through the use of the R package factoextra (https://cloud.r-project.org/package=factoextra). The PCA analysis was used to identify any sample cluster associated with the first two principal components. After the reads were normalized using TPM (Tags Per Million) for each of the genes found in the samples, an in-house script (Sogen) was employed to identify the next-generation sequence data as well as the genomic annotations. Finally, the pheatmap package accompanying the R package was employed to carry out clustering of the data that was based on Euclidean distance.

## Results

### Identification of sepsis-associated AS events from sepsis patients and healthy controls (Fig. 1)

**Fig. 1:**
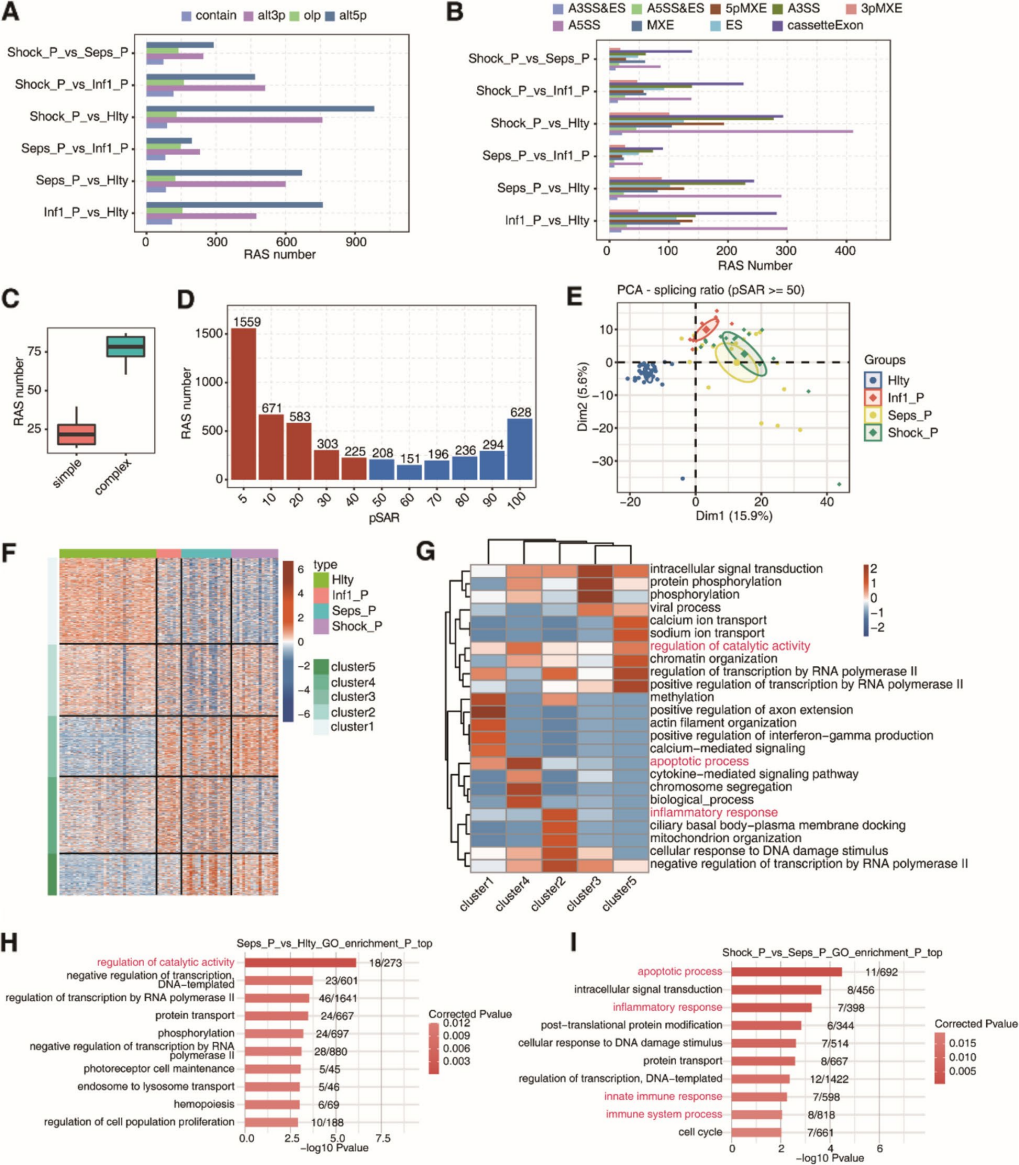
**A** Bar plot that indicates the number of regulated AS (RAS) events that were detected using SUVA among healthy control (Hlty), uncomplicated infection (Inf1_P), sepsis (Seps_P), and septic shock (Shock_P) groups. **B** Splice junctions (SJs) that comprise RAS events, as detected using SUVA. The SJs were annotated based on the types of classical AS events. The numbers for each type of classical AS event are illustrated as bar plots. **C** Box plot indicating the number of SUVA RAS events that contain the SJs that involve two or more classical splicing events (designated as complex) or involve the same classical splicing event (designated as simple). **D** Scatter plot indicating the RAS events with different pSAR. RAS events in which the pSASR (reads as a proportion of the SUVA AS events) >= 50% are labeled. **E** Principal component analysis (PCA) of the RAS event splicing ratio where pSAR >= 50%. The samples were clustered as tumor or normal. Each group’s ellipse represents the confidence ellipse. **F** Heatmap of the splicing ratio across all RAS event samples where pSASR >= 50%. RAS events were clustered using K-means. **G** The five most highly enriched GO terms that were biological processes of RAS genes (RASG) in each cluster are shown as a heatmap. Colors indicate the scaled -log10 FDR for each term in the column direction. **H** GO analysis of RASG between sepsis and healthy samples. The top 10 most highly enriched GO terms that were biological processes are shown. **I** GO analysis of RASG between septic shock and sepsis samples with the top 10 most highly enriched GO terms that were biological processes are shown

To investigate the gene expression changes and the alterations in molecular function of alternative splicing in sepsis, we assessed the publicly available mRNA-seq data obtained from the NCBI GEO dataset (GSE154918). This dataset featured hospitalized patients who presented with a bacterial infection within the last 24 h. Participants were assigned to a sepsis group and non-complicated infection groups. In addition, healthy volunteers were recruited as controls. After identifying high-quality filtered reads, we performed SUVA analysis and identified significant various splicing events in each group. A total of 30,161 unique AS events were identified in sepsis when compared to healthy controls. In total, we identified nine types of AS events: A3SS, A5SS, ES, 3pMXE, 5pMXE, MEX, Cassetteexon and A5SS&ES. The highest proportion of AS events featured the A5SS type. A3SS&ES and A5SS& ES events were recognized as significant forms of AS but with the lowest proportion of event (Fig. 1A-B). Approximately 75% of RAS events involved complex splicing events (Fig. 1C). SAR was then used to identify the proportion of splicing events at the transcriptional level in whole genes, more than 60% of RAS events identified were secondary transcripts; the PSAR of 1559 RAS events was less than 5% (Fig. 1D).

Then the AS expression features from the other groups were assessed by completing a principal component analysis (PCA) that was based on the splicing ratio. It was determined that sepsis, mild infection, and control samples could be identified separately using the second component. In addition, the sepsis and sepsis shock samples clustered together (Fig. 1E). The different splicing patterns of DEGs in sepsis were displayed by Heatmap, thus indicating that the splicing pattern in sepsis may have higher levels of heterogeneity (Fig. 1F). In addition, functional cluster analysis demonstrated that the genes related to AS events were mostly enriched in terms of “regulation of catalytic activity,” “inflammatory response,” and “apoptotic response” (Fig. 1G). Furthermore, analysis using GO indicated that the AS genes showing differential expression were mostly enriched in the “regulation of catalytic activity” in the infection group when compared to the healthy group. Furthermore, “immune system process,” “innate immune response,” and “inflammatory response” were the most significantly enriched biological processes in the septic shock group when compared to the sepsis group (Fig. 1H-I). These results indicated that sepsis-specific AS genes were decidedly associated with critical biological processes closely related to sepsis.

### The detection of dynamic AS events in apoptosis-related genes in different groups

**Fig. 2:**
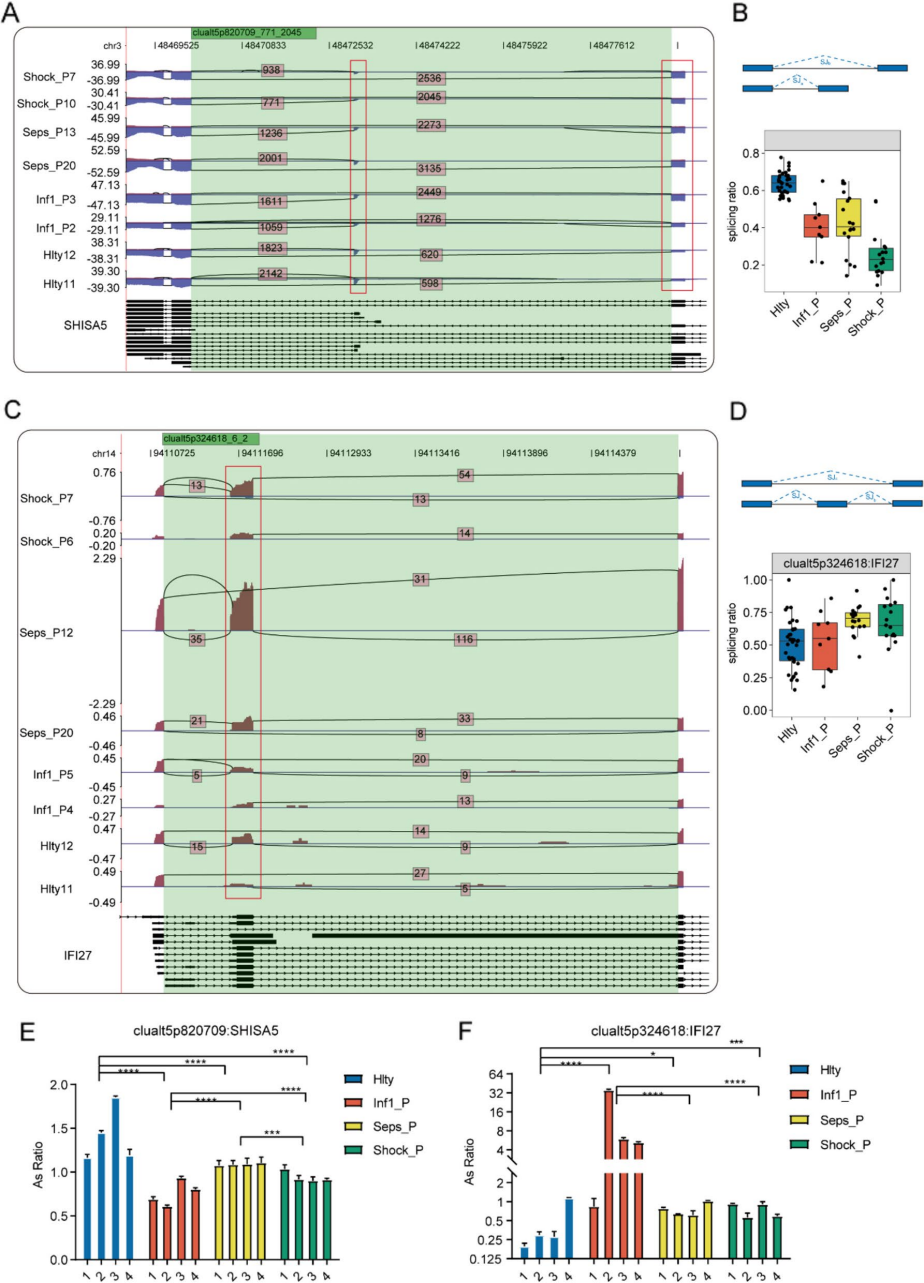
Dynamic alternative splicing was identified in apoptosis-related genes among different groups. **A** The distribution of the junction reads from SHISA5 in samples are visualized for the different groups. The numbers of SJ reads are included as labels with the splice junctions. Altered exons are marked using red boxes. **B** The upper panel shows a slicing events model. The lower panel shows a boxplot indicating the splicing ratio profile across the splicing events from *SHISA5* from the 79 samples shown in (**A**). **C** Visualization of the distribution of junction reads for *IFI2*7 in samples from the various study groups. The splice junctions are labelled with the number of SJ reads. Altered exons are shown in the red box. **D** The upper panel shows a splicing events model. The boxplot shown in the lower panel indicates the splicing ratio profile across the splicing events from *IFI27* from the 79 samples, as seen in (**C**). The validation of *IF127* and *SHISHA5* is shown in (**E**–**F**)

Next, we combined the results of the analysis of the AS events pathway with the results from the RNA-seq expression. We observed that *IFI27* and *SHISA5*, both apoptosis-related genes, were controlled by the 5pMXE AS pattern and showed significant differential expression levels in sepsis when compared to the healthy group and the infection group (Fig. 2A-D). Furthermore, the frequency of exon retention events was higher in septic shock than sepsis, thus indicating that exon retention events were closely related to the severity of sepsis. Importantly, in sepsis patients, the AS ratio of *IFI27* and *SHISA5* were significantly different when comparing the healthy group and infection group. Collectively, these results indicated that cell apoptosis-related genes were globally activated and showed distinct AS patterns which may be potential targets for sepsis.

### Differentially expressed RNA binding proteins (DE RBPs) in sepsis patients and healthy donors

**Fig. 3:**
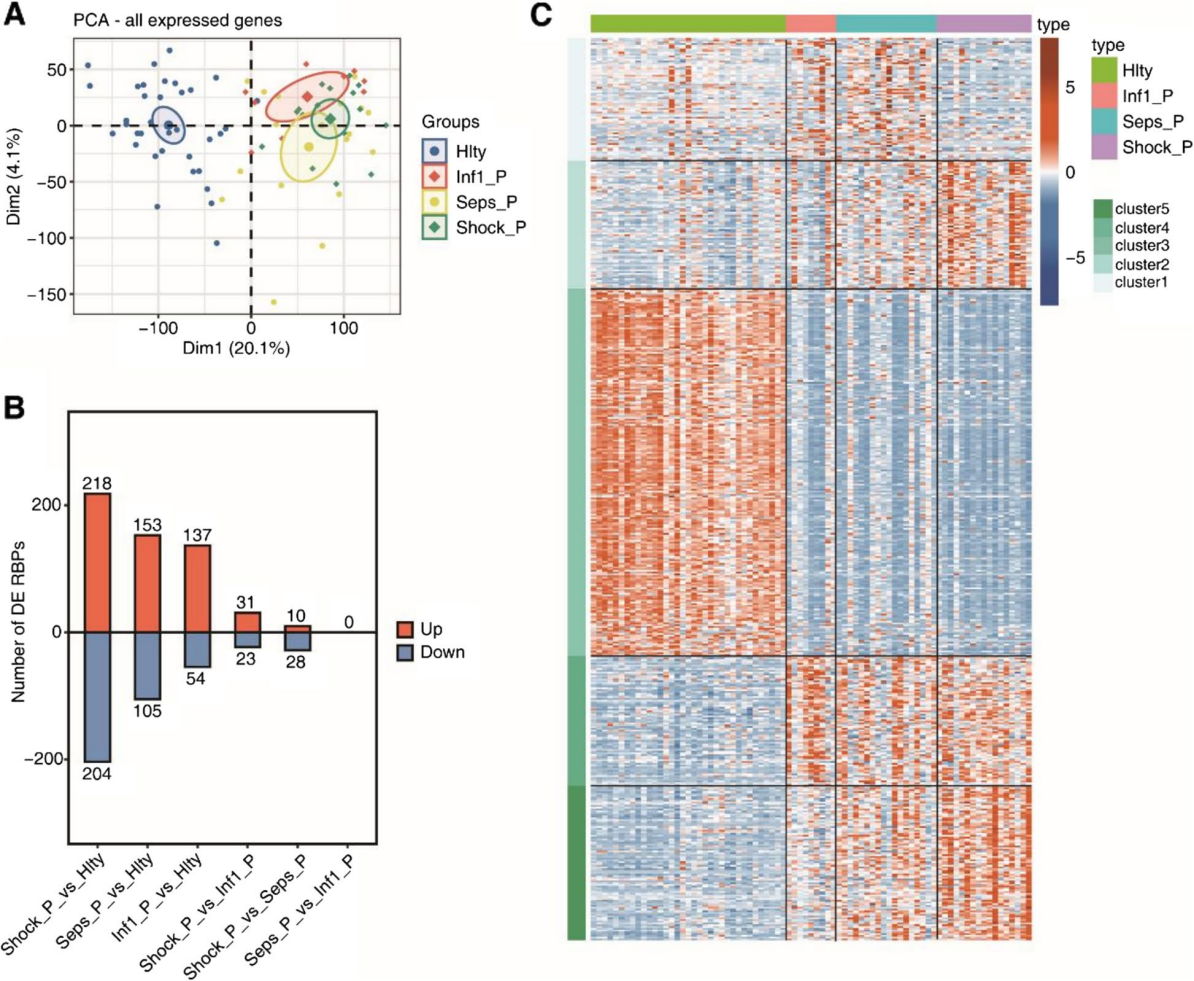
Differentially expressed RNA binding proteins (DE RBPs) in sepsis patients and healthy donors. **A** Principal component analysis (PCA) utilizing the FPKM values of all the expressed genes. The ellipse associated with each group represents the confidence ellipse. **B** The numbers of DE RBPs among the different groups are shown as a bar plot. Up: Up-regulated; Down: Down-regulated. **C** Expression heatmap of all 514 significant DE RBPs among different groups. DE RBPs were clustered using K-means

Analysis of the DEGs revealed statistical significance relating to RNA binding proteins in different groups. The healthy controls and patients were clearly differentiated based on gene expression ratio in PCA. However, the mild infection, sepsis, and sepsis shock groups could not be clearly separated (Fig. 3A). The number of RBPs was related to the severity of infection; the proportion of differentially expressed RNA binding proteins was highest in the sepsis shock; this was significantly higher than all the other groups (Fig. 3B). The differential expression patterns of RBPs in each group are shown as a heatmap in Fig. 3C including RBPs showing significant differences between the disease group and the healthy group; there were no statistically significant differences between the septic shock and sepsis group (Fig. 3C). The distinct expression of RBPs was clearly associated with the pattern of AS events, which then affected the cellular biological process and played a role in the pathophysiology of sepsis.

### AS events coordinate with RBP genes in sepsis patients and healthy donors

**Fig. 4:**
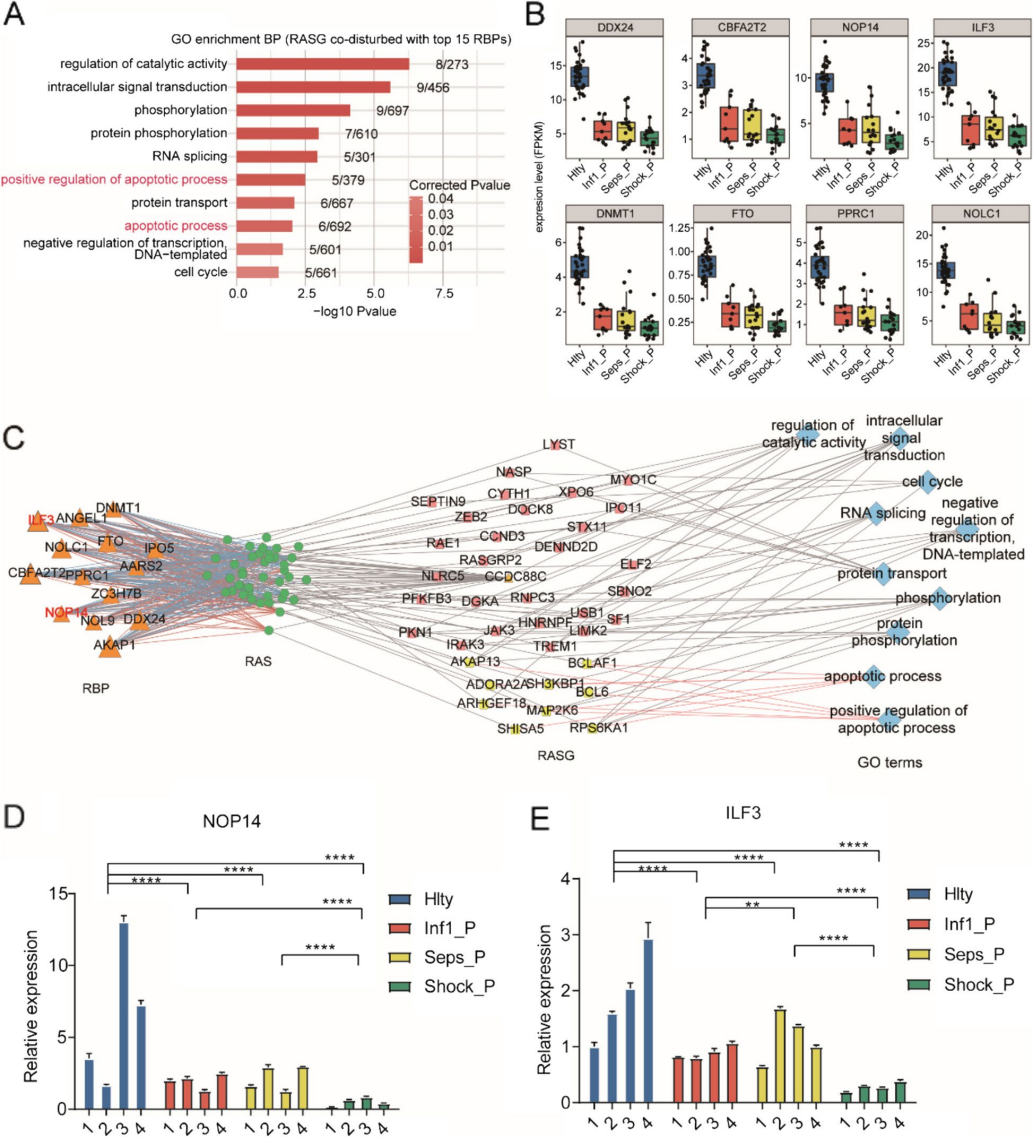
Alternative splicing coordinates with RBP genes in sepsis patients and healthy donors. **A** Enriched GO biological processes showing the top 15 genes showing variable splicing events that were associated with differentially expressed RBP genes. **B** A boxplot is shown that indicates the co-disturbed RBP expression profiles in sepsis patients and healthy controls. **C** The co-disturbed network is shown that was constructed between the DE RBP expression values and the splicing ratio for the RAS events (pSAR >= 50%) using 79 samples (sepsis patients and healthy controls). Samples were retained that exhibited a Pearson’s correlation >= 0.7 and a *P*-value <= 0.01. Co-disturbed RAS and RASG that were involved in the most highly enriched GO terms (furthest right) are indicated in blue. The validation of NOP14 and ILF3 in sepsis patients (**D**-**E**)

**Fig. 5:**
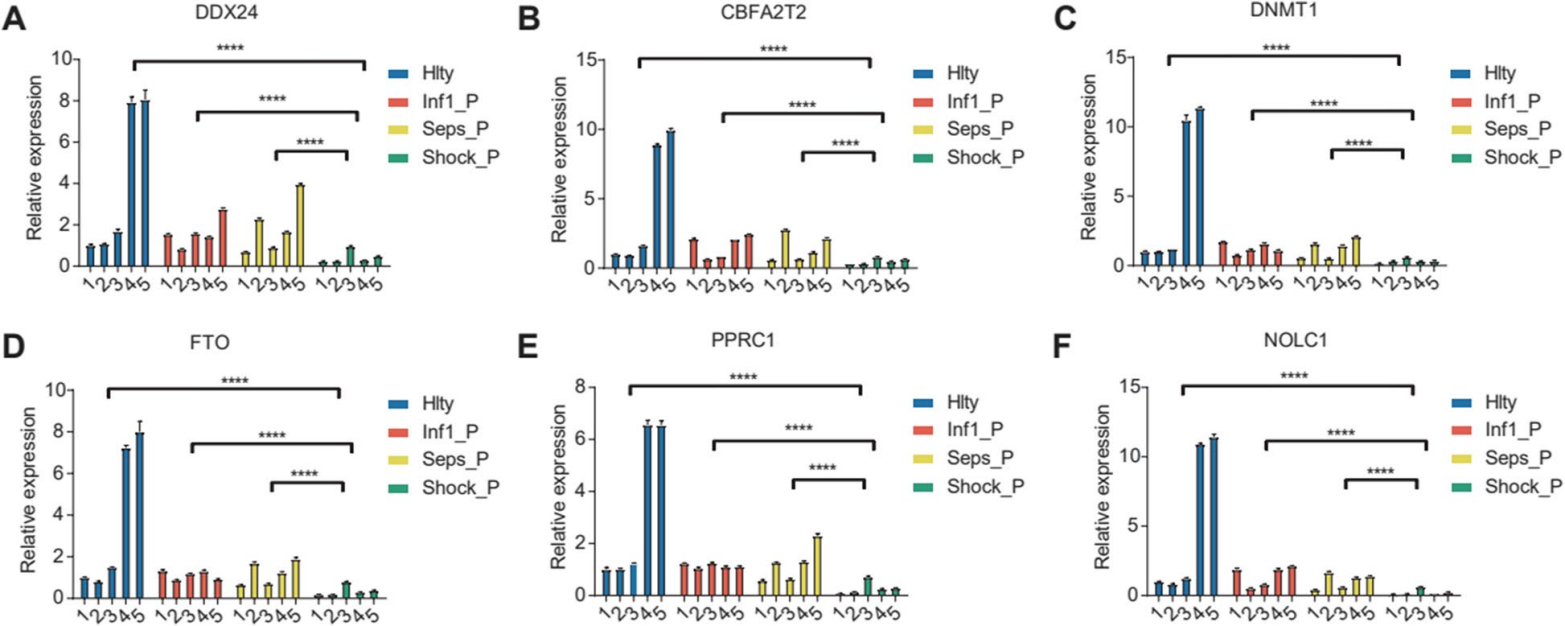
The validation of DDX24, CBFA2T2, DNMT1, FTO, PPRC1, NOLC1in sepsis patients (**A**-**F**)

The significant interactions between AS events and RBP genes were particularly enriched in GO biological process terms related to cellular apoptosis (Fig. 4A). The expression levels of DDX24, CBFA2T2, NOP, ILF3, DNMT1, FTO, PPRC1, and NOLC1 that were present in the sepsis group were decreased significantly compared to the expression observed in the control group (Fig. 4B). To validate the changes in expression observed for the sepsis-associated RBP genes in the GSE154918 RNA-seq data, the eight RBPs and two highly co-expressed AS-related genes from the coexpression network (Fig. 4C) underwent qRT-PCR validation using the blood samples that were obtained from the sepsis patients as well as the normal controls. In particular, DDX24, CBFA2T2, NOP, ILF3, DNMT1, FTO, PPRC1, and NOLC1 were co-expressed and featured the most AS events in the immune cell apoptosis network (Figs. 4D-E and 5A-F). The results obtained from the qRT-PCR were similar to the results from the RNA-seq analysis of GSE154918. Thus, the expression levels of the eight RBPs (DDX24, CBFA2T2, NOP14, ILF3, DNMT1, FTO, PPRC1, and NOLC1) were altered in the patients with sepsis might potentially be useful as novel biomarkers as well as targets to facilitate the diagnosis and management of sepsis. Together, these findings indicated that RBPs might carry out critical roles in regulating the associated genes by alternative splicing events that are related to cellular apoptosis in sepsis.

## Discussion

Most septicemia-related deaths arise from the death of a large number of immune effector, thus severely impairing the immune response and creating a sustained immunosuppressive state [16, 17]. In particular, the apoptosis of lymphocytes, such as, innate, immune and adaptive immune cells is associated with an elevated risk for secondary infections as well as a poor prognosis in sepsis patients [18]. Therefore, specific apoptotic and anti-apoptotic pathways may represent attractive targets for sepsis [19]. In general, mechanisms underlying the expression of genes in sepsis may involve circular RNAs (circRNAs) [20], a stable form of non-coding RNA [21]; often these act as sponges for miRNA [22]. These sponges carry out critical functions in gene transcription and gene expression [23] and are known to engage in a range of cellular events, including differentiation, proliferation, apoptosis, and autophagy [24, 25]. Furthermore, in sepsis patients, circRNAs are known to be more abundant in immune cells than in healthy subjects [26]. The identified biological functions, as well as other characteristics, suggest that transcriptional and post-transcriptional regulation might carry out essential functions in the pathophysiology of sepsis [27, 28].

AS as well as mRNA isoforms participate in critical functions in nearly every cell process and pathway. Therefore, it was not unexpected that both were determined to be essential in effective antiviral responses [29]. AS events have been documented to participate in making fine adjustments to both responses. As an example, the Toll-like receptor signaling pathways, which are associated with innate immunity, are known to be controlled by AS events as well as alternative polyadenylation [30]. AS events also carry out critical functions in the activation of lymphocytes. The activation of T- and B-cells can considerably alter gene expression and AS events [31]. The changes in AS in these cells are not well-understood. However, one recent study profiled the B-cell transcriptional response to stimuli that activate B-cells [31]. The results from that study revealed that AS events, in particular the use of exons, affect numerous genes, with pronounced enrichment occurring in genes associated with functions in signaling and receptors [32].

Our study showed that the Cassetteexon, A5SS, A3SS were the most variable splicing events in sepsis. Approximately 75% of RAS events are complex splicing events. The splicing patterns in sepsis may have higher levels of heterogeneity, thus indicating the complexity of splicing regulation in sepsis. Our PCA plot also demonstrated that the mRNA AS events themselves could represent a solid comprehensive signature for detecting sepsis. As reported earlier, the alternative splicing of sphingomyelin phosphodiesterase1(SMPD1) functions as a negative regulator of acidic sphingomyelinase (ASM) activity. The activity of acidic ASM or SMPD carries out essential functions associated with the development of immune responses and organ failure in sepsis [33, 34]. In addition, a reduction in intron 5 retention is known to be a risk factor for severe sepsis and septic shock [35]. It has been reported that the polymorphic variants associated with exon 8 that are located at the 3´-UTR of the *HLA-G* gene are connected with septic shock that can occur in patients that are critically ill [36]. These results suggest that alternative splicing involves regulation of the immune/inflammatory response in sepsis, which might have aa crucial function in the mechanism of sepsis.

In this study we identified various AS patterns in genes related to cell apoptosis, including *SHISA5, IFI27, HTT, MCL1, NR3C1, DELE1, NUDT2, FHIT.* All of these genes showed significantly different RNA expression levels in the different groups. Interestingly, 5pMXE and 3pMXE were the most common events. As described earlier, the single-pass ER transmembrane protein SHISA5 has been reported to serve as a novel negative regulator of the processes underlying constitutive autophagy [36]. This finding is supported by the fact that SHISA5 deficiency alone is known to be sufficient to induce autophagy [37]. SHISA5 has been demonstrated to be a substrate in autolysosomal proteolysis, which is removed eventually from the site of action during the process of autophagy [38]. In addition, IFI27 was previously reported to be robustly associated with Th2 cells and Th1 cells, as well as aDC [38]. Furthermore, IFI27 has been shown to correlate positively with viral load, but it correlates negatively with counts of CD4 cells [39]. It is possible that IFI27 might participate in the mechanisms underlying immunodeficiency, and the expression of IFI27 might be associated with sepsis exacerbation, therefore representing a potential therapeutic target. These results indicated that the 5pMXE alternative splicing pattern of SHISA5 and IFI27 might be important targets of the immune cell apoptosis process in sepsis. Furthermore, skipping exon 6 of the *FAS* gene, which encodes the transmembrane domain, produces a soluble protein [39]. On the other hand, when this exon is included, a membrane receptor is produced, which triggers signaling pathways associated with cell death [40]. These findings indicate that the distinct AS patterns involved in sepsis pathophysiology might display essential functions in immune cell apoptosis in sepsis.

RNA-binding proteins (RBPs) are known to be crucial effectors in the expression of many genes [41] and are involved in the regulation of nearly every aspect of RNA functionality, including transcription, splicing, modification, intracellular trafficking, translation, and decay [42, 43]. Importantly, gene expression differences between sepsis survivors and non-survivors have been detected previously; furthermore, multiple genes related to immune function were poorly expressed in non-survivors [44]. This study has demonstrated that RBP expression was substantially different among the different study groups and clearly separated the sepsis group from the infection and healthy groups. Interestingly, the number of RBPs decreased with increasing severity of sepsis. The differential expression of RBPs during the process of sepsis may lead to differences in AS, thus affecting various aspects of cell function. GO analysis of the DE RBPs in sepsis identified terms that were mostly enriched in the immune/inflammatory response; we identified significant differences in 8 down-regulated RBPs in sepsis, including DDX24, CBFA2T2, NOP, ILF3, DNMT1, FTO, PPRC1, NOLC1. These changes indicate that the distinct expression levels of RBPs related to immune/inflammatory genes play important roles in the molecular mechanisms underlying the pathophysiology of sepsis.

The human nucleolar protein 14 (NOP14) gene has been reported previously to be located on chromosome 4p16.317 and is a key gene in the process of sepsis. This gene might be associated with pre‑18S rRNA processing that occurs during sepsis, or it might be involved in the inflammation that takes place during sepsis, acting through the regulation of miRNA‑2909 expression [45]. EMG1 and NOP14 are known to be members of a family of repressed environmental stress response (ESR) genes, which also includes genes that encode ribosomal proteins (RPs) as well as additional proteins known to be associated with RNA metabolism and the synthesis of proteins [46]. Furthermore, the enhancer binding factor 3 (ILF3) functions as a stable heterodimeric complex to stabilize mRNAs and regulate gene expression [47]. As reported previously, miR-215-5p expression is protective in inflammation injury that occurs in sepsis caused by H9c2 by targeting ILF3 and LRRFIP1 [48]. DDX24 is a DEAD-box helicase whose role in cells is largely unknown. However, DDX24 is capable of binding ssRNA and dsRNA. Interestingly, it has been shown to negatively affect RLR-dependent innate immune activation through several mechanisms [49]. DDX24 has been shown to compete with RIG-I for VSV RNA binding through its ability to bind RNA, exerting a direct inhibitory effect on viral perception [50]. At the same time, the qRT-PCR results of this study showed that the expression of DDX24 in the sepsis group was significantly lower than that in the healthy group, so we speculated that it plays a protective role in the innate immune response in sepsis through negative regulation.DNA methyltransferases1(DNMT1) -mediated DNA methylation is involved in many human diseases by affecting many types of cellular processes, including cell growth, cell cycle progression, metastasis, apoptosis, development, and tumorigenesis [51]. Fubing Ma et al. report that DNMT1-mediated increased DNA methylation plays a key role in LPS-induced sepsis by regulating the SMAD2/DNMT1/miR-145 negative regulatory loop [52]. The first discovered RNA demethylase obesity-associated protein (FTO) [53], involved in cell proliferation, apoptosis, cell cycle, migration, invasion, drug resistance and other processes [54]. As an oncogene, FTO promotes IDH mutations through the FTO/MYC/CEBPA signaling pathway, which in turn leads to tumorigenesis [55]. The human fragile histidine triad (FHIT) gene is a tumor suppressor gene, and heterozygous deletion (LOH), homozygous deletion, and abnormal expression of the FHIT gene have been implicated in several types of human malignancy [56]. In addition, FHIT has been reported to increase mitochondrial calcium release and promote apoptosis [57].The mechanism of action of FTO, NUDT2 and FHIT in sepsis is not clear, and we speculate that FTO, NUDT2 and FHIT may promote the development of sepsis by participating in apoptosis, and the mechanism needs further study. Collectively, these results suggested that genes encoding RBPs, such as DDX24, NOP, ILF3, DNMT1, FTO, PPRC1, NOLC1,represent novel targets for the molecular mechanisms that regulate sepsis and may be involved in the immune response by regulating AS events in key genes that are associated with cellular apoptosis.

Our study has some limitations that should be considered, even though we detected significant differences in AS in sepsis compared with a healthy group. However, we are still unclear whether changes in AS are related to certain bacterial categories. The factors that exert impact on AS in sepsis needs further research. Similarly, we need to investigate the mechanisms and clinical outcomes of ILF3 regulation on crucial genes in sepsis.

## Conclusion

In this study, we describe the characteristic of AS and characterize the function of genes related to AS in sepsis. Furthermore, we identified the significant role of RBPs in the progression of sepsis. In addition, the functional pathways of RASG were related to the apoptosis pathway, the regulation of catalytic activity, and the positive regulation of apoptosis. We also generated a functional path diagram for the RBP-RAS-RASG network, representing a novel RBP-based post-transcriptional network that links sepsis progression and immunomodulation within the sepsis microenvironment. These transcripts, in particular, encode secretory factors that not only limit the metastasis of sepsis, but also remodel the sepsis immune microenvironment towards immune function suppression. Alterations in splicing factors, including transcriptional alteration, and their functional impactions in sepsis development, have been extensively studied.

## Data Availability

All data generated or analyzed during this study have been included in this published article. The datasets supporting the results of this article are available in the NCBI Gene Expression Omnibus and are accessible through the GEO series accession number (GSE154918).
